# Cumulative Lead Exposure and Tooth Loss in Men: The Normative Aging Study

**DOI:** 10.1289/ehp.0900739

**Published:** 2009-06-15

**Authors:** Manish Arora, Jennifer Weuve, Marc G. Weisskopf, David Sparrow, Huiling Nie, Raul I. Garcia, Howard Hu

**Affiliations:** 1 Environmental and Occupational Medicine and Epidemiology, Harvard School of Public Health, Boston, Massachusetts, USA; 2 Population Oral Health, Faculty of Dentistry, University of Sydney, Sydney, New South Wales, Australia; 3 Rush Institute for Healthy Aging, Rush University Medical Center, Chicago, Illinois, USA; 4 Department of Environmental Health, Harvard School of Public Health, Boston, Massachusetts, USA; 5 The Normative Aging Study, Veterans Affairs Boston Healthcare System, Boston, Massachusetts, USA; 6 Department of Medicine, Boston University School of Medicine, Boston, Massachusetts, USA; 7 Department of Health Policy and Health Services Research, Henry M. Goldman School of Dental Medicine, Boston University, Boston, Massachusetts, USA; 8 Department of Environmental Health Sciences, University of Michigan School of Public Health and Medicine, Ann Arbor, Michigan, USA

**Keywords:** aging, blood lead, bone lead, KXRF, tooth loss

## Abstract

**Background:**

Individuals previously exposed to lead remain at risk because of endogenous release of lead stored in their skeletal compartments. However, it is not known if long-term cumulative lead exposure is a risk factor for tooth loss.

**Objectives:**

We examined the association of bone lead concentrations with loss of natural teeth.

**Methods:**

We examined 333 men enrolled in the Veterans Affairs Normative Aging Study. We used a validated K-shell X-ray fluorescence (KXRF) method to measure lead concentrations in the tibial midshaft and patella. A dentist recorded the number of teeth remaining, and tooth loss was categorized as 0, 1–8 or ≥ 9 missing teeth. We used proportional odds models to estimate the association of bone lead biomarkers with tooth loss, adjusting for age, smoking, diabetes, and other putative confounders.

**Results:**

Participants with ≥ 9 missing teeth had significantly higher bone lead concentrations than those who had not experienced tooth loss. In multivariable-adjusted analyses, men in the highest tertile of tibia lead (> 23 μg/g) and patella lead (> 36 μg/g) had approximately three times the odds of having experienced an elevated degree of tooth loss (≥ 9 vs. 0–8 missing teeth or ≥ 1 vs. 0 missing teeth) as those in the lowest tertile [prevalence odds ratio (OR) = 3.03; 95% confidence interval (CI), 1.60–5.76 and OR = 2.41; 95% CI, 1.30–4.49, respectively]. Associations between bone lead biomarkers and tooth loss were similar in magnitude to the increased odds observed in participants who were current smokers.

**Conclusion:**

Long-term cumulative lead exposure is associated with increased odds of tooth loss.

Although the successful implementation of public health policies has lowered environmental lead exposure in the United States, individuals previously exposed to this toxicant remain at risk because of endogenous release of lead stored in their skeletal compartments (Hu et al. 1998; Vig and Hu 2000). The majority of lead absorbed into the body is incorporated into bones from which it may interchange with other tissues. Bone lead biomarkers have been successfully applied to estimate long-term cumulative environmental lead exposure and have been linked with a number of adverse health effects, including neurologic impairment, hypertension, and renal dysfunction (Glenn et al. 2003; Navas-Acien et al. 2007; Shih et al. 2007; Tsaih et al. 2004; Weaver et al. 2005; Weisskopf et al. 2007). Although emerging evidence suggests that lead exposure may increase the risk of some oral diseases, no study has previously investigated whether bone lead levels are associated with tooth loss.

Lead is known to disrupt several cellular and molecular pathways that are relevant to the health of oral tissues. Lead can alter both humoral and cell-mediated immunity, and recent evidence suggests that lead exposure may affect regulation of inflammatory cytokines in occupationally exposed workers (Singh et al. 2003; Valentino et al. 2007). Furthermore, lead may induce oxidative stress in a number of tissues and organs, including salivary glands (Abdollahi et al. 2003; Ahamed and Siddiqui 2007). These effects of lead are pertinent to common oral diseases including dental caries and periodontitis, which have previously been associated with tooth loss (Eklund and Burt 1994; Locker et al. 1996).

Loss of natural teeth is an important public health issue, with approximately 25% of American adults ≥ 60 years of age experiencing complete tooth loss (Beltran-Aguilar et al. 2005). Poor oral health, while in itself associated with a decline in quality of life (Brennan et al. 2008; Naito et al. 2006), is also linked to adverse alterations in diet and increased risk of cardiovascular disease, cancer, and a number of other systemic conditions (Bahekar et al. 2007; Michaud et al. 2007; Walls et al. 2000). Therefore, it is increasingly important to establish the risk factors, including the environmental determinants, of oral health. In the present study we investigated the association of long-term cumulative lead exposure, as determined by bone lead concentrations, with loss of natural teeth in a cohort of older U.S. males.

## Methods

### Study participants

Participants were from the Normative Aging Study, a longitudinal study established by the Veterans Administration in 1963 (Bell et al. 1972). Between 1963 and 1968, a total of 2,280 male volunteers 21–80 years of age were enrolled in the study. Exclusion criteria included history of heart disease, hypertension, diabetes, cancer, peptic ulcer, gout, recurrent asthma, bronchitis, or sinusitis at baseline, which was approximately 25 years prior to the measurement of lead bio-markers and dental health variables. Between 1992 and 1994, a total of 899 participants were examined, and data on number of natural teeth remaining were available for 547 participants. For the present study, we included 333 of these participants who also had valid bone lead measurements. All participants gave informed consent, and the study was approved by the Human Research Committees of Brigham and Women’s Hospital, Harvard Medical School, and the Department of Veterans Affairs Boston Healthcare System.

### Bone and blood lead measurements

Beginning in 1991, we measured bone lead at two sites (tibial midshaft and patella) with a K-shell X-ray fluorescence (KXRF) instrument (ABIOMED, Inc., Danvers, MA, USA). The tibia and patella are targets of choice during KXRF measurements, as they consist primarily of cortical and trabecular bone, respectively, and are representative of the two main bone compartments in the human body. Methodologic details of the KXRF instrument have been described elsewhere (Burger et al. 1990; Hu et al. 1990). In brief, the instrument used a ^109^Cd γ-ray source to provoke the emission of fluorescent photons from target tissue, which were then detected, counted, and arrayed on a spectrum. A net lead signal is determined in micrograms lead per gram of bone mineral. The instrument also provides an estimate of the uncertainty associated with each measurement that is derived from a goodness-of-fit calculation of the spectrum curves. Blood samples were analyzed by graphite furnace atomic absorption spectroscopy (ESA Laboratories, Chelmsford, MA, USA). The instrument was calibrated every 21 samples with National Bureau of Standards blood lead standards materials. Ten percent of the samples were run in duplicate; at least 10% of analyses were controls and 10% were blank. Additional details of blood lead analyses have been described elsewhere (Hu et al. 1996). For six participants who had blood lead concentrations below the detection limit (DL) of 1 μg/dL, we imputed a value equaling 
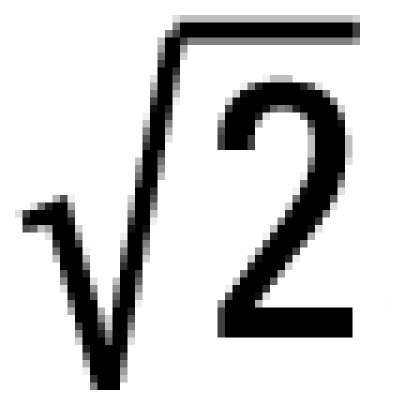
 (i.e., 0.71 μg/dL).

### Tooth loss and covariates

From 1992 to 1994, a trained dentist undertook dental examinations and recorded the number of natural teeth present on a subgroup of Normative Aging Study participants. We grouped participants into three categories of tooth loss: 0, 1–8 or ≥ 9 missing teeth. We selected these categories because losing ≥ 8 natural teeth, other than third molars (i.e., having < 20 teeth remaining), is indicative of reduced chewing ability (Helkimo et al. 1978; Ueno et al. 2008). Using a questionnaire, we also recorded brushing frequency. Participants were asked to indicate if they were current smokers (yes vs. no) or past smokers (yes vs. no) on a questionnaire. Those who responded “no” to both items were classified as never-smokers. We calculated pack-years by multiplying the reported average number of cigarettes smoked daily by the number of years smoked and dividing this product by 20 (cigarettes per pack). We classified participants as having uncontrolled diabetes if they had a fasting plasma glucose level ≥ 126 mg/dL or having controlled diabetes if they had a physician diagnosis of diabetes and fasting plasma glucose level < 126 mg/dL.

### Statistical analysis

We analyzed the distribution of blood, tibia, and patella lead concentrations within key subject characteristics. To make full use of the three categories of tooth loss, we used ordinal logistic regression— specifically, proportional odds models—to estimate the association of the lead biomarkers with tooth loss. Using this type of analysis, we simultaneously modeled two logits (or *k*-1 logits for *k* ordinal categories of an outcome) in cumulative fashion. That is, for the exposures *X* in relation to the categories of missing teeth *Y*:


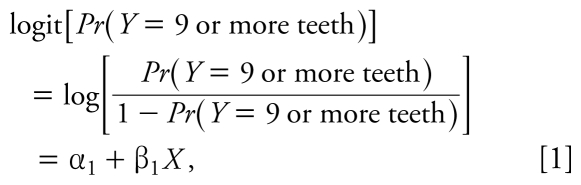



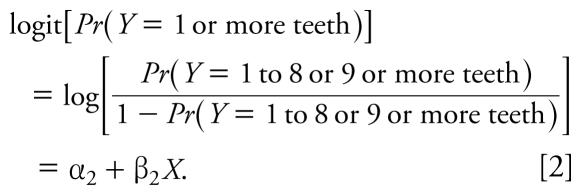


By using proportional odds models, we assume that the odds of an event, given a set of exposure values, are the same across both equations, with the exception of the intercept factors (i.e., the proportions). This means that β_1_ = β_2_, providing a common odds ratio (OR) for a given exposure *x_j_*, exp(β*_j_*), across all progressively encompassing degrees of tooth loss. For instance, using this approach we can estimate the OR for the highest tertile of tibia lead, with the lowest tertile as the reference group. The OR for the outcome of ≥ 9 missing teeth versus 1–8 missing teeth or 0 missing teeth combined [exp(β_1, highest Pb_)] is the same as the OR for the outcome of 1–8 missing teeth or ≥ 9 missing teeth combined versus 0 missing teeth [exp(β_2, highest Pb_)]. We confirmed the proportionality assumption for all models (Hosmer and Lemeshow 2004).

In our analyses, we used tertiles of the lead biomarkers and estimated separate models for lead concentrations in tibia, patella, and blood. We incorporated key variables that have been previously associated with environmental lead exposure and tooth loss: age (years), education (< high school, high school, technical training/some college, or college graduate or higher), smoking status (current, past, or never-smoker), pack-years of smoking, and diabetes (yes or no). We also considered the effects of including questionnaire-recorded tooth brushing frequency and dietary calcium intake. However, because these variables did not alter the relationship between the lead biomarkers and tooth loss and were not significant predictors of tooth loss, we excluded them from our final analyses. To further examine the effects of socioeconomic factors on our analyses, we estimated the association of lead biomarkers and tooth loss in separate categories of education—those with high school or lower education versus participants who had technical, college, or higher training. Our sample was racially homo genous, with all but six of our participants identifying themselves as white Americans. To confirm that race was not a confounding factor in our analyses, we excluded the six non white participants from the analyses and observed no appreciable change in the results. We therefore included all participants in our final analyses. We also compared key characteristics of Normative Aging Study participants included in our study with those excluded because of missing data on dental or lead biomarker variables. For all data analyses, we used SAS version 9.1 (SAS Institute Inc., Cary, NC, USA).

## Results

Our participants had a median age of 67 years (range, 50–94 years). Approximately 13% (*n* = 44) had not lost any of their natural teeth, and 38% (*n* = 125) had lost ≥ 9 teeth. Compared with those excluded from our analyses because of missing data on tooth loss or lead biomarkers, our study participants were younger by an average of 1.1 years (*p* = 0.05), had a greater proportion of never-smokers (31.2% vs. 23.9%; *p* = 0.05), and had higher fasting plasma glucose levels (114.63 vs. 106.26 mg/dL; *p* = 0.0001). However, there were no significant differences between these groups in terms of pack-years of smoking, educational attainment, or physician diagnoses of diabetes.

Participants with ≥ 9 missing teeth were more likely to be older and have less education than those with no tooth loss. They were also more likely to be current smokers and have experienced more than 10 pack-years of smoking. Men with ≥ 9 missing teeth had the highest bone lead levels, but we found no significant difference in blood lead concentrations between the three categories of tooth loss (Table 1). As has been described earlier in this cohort (Schaumberg et al. 2004), bone lead concentrations were higher in older participants. Of all the lead biomarkers, patella lead levels were most closely associated with the smoking variables; current and past smokers and those with > 10 pack-years of smoking history had higher patella lead concentrations. Men who had not completed high school had the highest bone and blood lead concentrations.

Using multivariable-adjusted proportional odds models, we found that participants in the highest tertile of tibia lead concentration had approximately three times the odds of having experienced an elevated degree of tooth loss (≥ 9 vs. 0–8 missing teeth or ≥ 1 vs. 0 missing teeth) as those in the lowest tertile of tibia lead concentration [OR = 3.03; 95% confidence interval (CI), 1.60–5.76) (Table 2). A similar association was also observed for participants in the highest tertile of patella lead (OR = 2.41; 95% CI, 1.30–4.49). Blood lead concentrations, however, were not significantly associated with tooth loss (OR = 0.88; 95% CI, 0.52–1.50).

To put the results for lead exposure in the context of more established risk factors for tooth loss, we estimated the relationship of cigarette smoking with tooth loss. Compared with participants who never smoked cigarettes, current smokers showed increased odds of tooth loss in the multivariable-adjusted models that included tibia lead concentrations (OR = 3.23; 95% CI, 0.99–10.48). Similar associations were observed for models that included patella or blood. Moreover, in all models for both current and former smokers, a 10 pack-year increment in smoking was associated with 21–26% increase in odds of tooth loss (e.g., for the model including tibia lead, OR = 1.21; 95% CI, 1.07–1.36). Additionally, there was an increase of 6–8% in the odds of experiencing tooth loss per year increment in age in the three separate models that adjusted for lead concentrations in tibia, patella, or blood. Participants with less than high school education and those with diabetes (controlled or uncontrolled) also had increased odds of tooth loss; however, these associations were not statistically significant.

When we stratified our analyses by level of educational attainment, we observed that the association of bone lead biomarkers with tooth loss was similar among the different categories of education. The ORs (95% CIs) of tooth loss (≥ 9 vs. 0–8 missing teeth or ≥ 1 vs. 0 missing teeth) for participants with technical, college, or higher training were 3.35 (1.44–7.78) and 2.87 (1.24–6.61) for the highest tertile of tibia and patella lead concentrations, respectively. Similarly, for men with education levels of high school or lower, the ORs of tooth loss were 3.29 (1.22–8.84) and 2.37 (0.87–6.43) for the highest tertile of tibia and patella lead concentrations, respectively.

## Discussion

In our study, men with elevated bone lead levels had approximately three times the odds of having experienced an elevated degree of tooth loss compared with those participants who were in the lowest tertile of bone lead concentrations. This association showed a clear trend across tertiles of bone lead biomarkers and remained significant when we adjusted for a number of confounding factors, including age, smoking, and diabetes. Moreover, blood lead levels were not associated with tooth loss, suggesting that bone lead concentrations are a better indicator of the risk of tooth loss posed by cumulative long-term environmental lead exposure.

Tooth loss is a multifactorial disease and is influenced by numerous sociodemographic and lifestyle factors (Beltran-Aguilar et al. 2005). Notably, environmental lead exposure has been linked to dental caries and periodontal disease (Moss et al. 1999; Saraiva et al. 2007), two important causes of tooth loss. It is, therefore, possible that the rate of tooth loss over the 25- to 30-year period, from baseline to the early 1990s, was greater in those participants with higher lead levels. Although the biological mechanisms linking lead exposure and dental disease are not completely clear, it has been suggested that lead may disrupt salivary gland function, thereby increasing the risk of dental caries (Watson et al. 1997). Studies on rats have shown that exposure to lead during the prenatal and peri natal periods resulted in significantly higher levels of dental caries and markedly reduced salivary flow rate (Watson et al. 1997). Similarly, adult rats exposed to lead through drinking water showed decreased salivary calcium and protein concentrations (Abdollahi et al. 1997). Although adult animals showed no change in salivary flow rate, increased lipid peroxidation and a decrease in total antioxidant capacity and thiol group levels in salivary gland tissue indicated the presence of lead-induced oxidative stress (Abdollahi et al. 1997).

The disruption of bone remodeling due to lead exposure has been suggested as a possible mechanism linking this toxicant with periodontal disease (Saraiva et al. 2007). That lead disrupts bone remodeling is supported by studies in laboratory animals where long-term lead exposure induced osteopenia (Gruber et al. 1997). Studies in children, however, suggest that this interaction is complex, and those exposed to lead may undergo accelerated bone maturation resulting in lower peak bone mass and, therefore, a greater risk of osteoporosis in older age (Campbell et al. 2004). Historical exposure to lead as reflected in bone lead concentrations may also be important in establishing susceptibility. The mechanisms behind the pathologic effects of lead on bone include the ability to promote inflammation, which is pertinent to perio dontal disease, where disruption of the host inflammatory response is considered an important factor in disease progression and subsequent alveolar bone loss. *In vitro* studies have reported that lead may affect the production of cytokines such as interleukin (IL)-2 and IL-10 and tumor necrosis factor-alpha (TNF-α) (Krocova et al. 2000). This finding is supported by a recent report of increased plasma IL-10 and TNF-α in occupationally exposed workers (Valentino et al. 2007).

In our multivariable-adjusted analyses, the association between bone lead biomarkers and tooth loss was similar in magnitude to the increase in odds observed among current smokers, indicating that long-term cumulative lead exposure may be a potentially important predictor of tooth loss. Like the participants in our study, the older members of the U.S. population, who were exposed to lead prior to the restrictions on its use in gasoline, paint, and other products, would have accumulated significant lead stores in their skeletal compartments that continue to place them at risk of a wide range of health effects. A number of studies have reported that bone lead levels increase with age (Hu et al. 1996; Kosnett et al. 1994; Wittmers et al. 1988), and it is possible that the increased risk of tooth loss due to accumulated lead may persist for many years to come.

Our study is limited by its cross-sectional design. However, bone lead concentrations reflect exposure over decades (Hu et al. 1998), and it is likely that much of the exposure in this cohort preceded some or all of the observed tooth loss. Furthermore, reverse causality, such that tooth loss could lead to increased bone lead levels, is unlikely. We have adjusted for a number of important potential confounders, including smoking. However, both lead exposure and tooth loss are influenced by a multitude of socioeconomic and environmental factors and, as in any observational study, the possibility of residual confounding due to unmeasured or mismeasured shared risk factors cannot be excluded. All of our study participants were male, with a majority identifying themselves as white Americans, limiting the generalizability of our results to the wider U.S. popu lation. Furthermore, in the present study, we analyzed data on a subgroup of participants recruited from the wider Normative Aging Study cohort. However, the self-selection of participants for dental examinations occurred independently from their bone lead measurements and is unlikely to bias the results of our study. Differential selection into the study on the basis of greater degrees of tooth loss and exposure to lead (the most likely pattern of selection) would only have underestimated the associations of lead biomarkers with tooth loss. All of our participants received dental care from private dental practitioners, and the clinical diagnoses and interventions leading to the removal of teeth would have been consistent with general population in the region. Our participants were younger and more likely to be never-smokers, making it unlikely that they were at greater risk of tooth loss than those who were not included in the study. Although the proportion of men with physician diagnosis of diabetes was similar in our participants and those not enrolled in our study, mean fasting plasma glucose levels were higher among those who were included in our analyses. Our study is strengthened by a large number of participants with detailed measures on a number of important covariates. Notably, we have used a validated KXRF method to measure *in vivo* lead concentrations in both the cortical and trabecular bone compartments.

To the best of our knowledge, this is the first epidemiologic investigation to show that bone lead levels are associated with tooth loss. Despite the decline in blood lead levels, lead exposure remains an important public health issue. The oral health implications of accumulated lead, however, are yet to be fully realized, and further work is needed to uncover the biological mechanisms underlying the association between bone lead and tooth loss.

## Figures and Tables

**Table 1:** Distribution of lead biomarkers [mean (95% CI)] within participant characteristics in the Normative Aging Study.

Characteristic	No.	Tibia lead (μg/g bone)	Patella lead (μg/g bone)	Blood lead (μg/dL blood)
Missing teeth				
0	44	15.1 (12.7–17.6)	23.2 (19.1–27.4)	5.3 (4.3–6.2)
1–8	164	21.0 (19.4–22.8)	32.0 (29.4–34.8)	6.2 (5.5–6.9)
≥ 9	125	24.9 (22.7–27.2)	37.0 (33.7–40.2)	6.3 (5.5–7.0)
Age (years)				
< 60	58	14.8 (12.8–16.7)	22.9 (19.8–26.0)	5.3 (4.4–6.2)
> 60–70	177	20.9 (19.2–22.6)	32.1 (29.6–34.6)	6.4 (5.7–7.1)
> 70	98	27.3 (24.6–29.9)	39.8 (35.8–43.7)	6.1 (5.4–6.8)
Education				
Less than high school	31	31.5 (24.7–38.2)	44.7 (35.1–54.3)	7.5 (5.7–9.3)
High school	118	22.7 (20.6–24.9)	34.9 (31.8–37.9)	6.1 (5.5–6.7)
Technical training/some college	75	22.1 (19.6–24.6)	32.9 (28.9–36.9)	5.8 (4.8–6.9)
College graduate or higher	96	17.8 (16.1–19.4)	26.7 (23.8–29.5)	6.1 (5.1–7.0)
Smoking status				
Never	104	21.1 (18.8–23.4)	30.8 (27.3–34.4)	6.1 (5.3–6.9)
Former	202	22.2 (20.5–23.9)	33.7 (31.3–36.2)	5.9 (5.3–6.5)
Current	24	19.6 (16.4–22.8)	33.4 (27.4–39.4)	7.7 (5.6–9.9)
Pack-years of smoking				
0	104	21.1 (18.8–23.4)	30.8 (27.3–34.4)	6.1 (5.3–6.9)
1–10	55	21.2 (16.6–25.8)	31.9 (26.1–37.6)	5.0 (3.9–6.0)
> 10	166	21.8 (20.3–23.3)	33.7 (31.3–36.0)	6.2 (5.6–6.8)
Diabetes				
No	274	20.9 (19.5–22.2)	31.8 (29.8–33.8)	6.2 (5.7–6.7)
Controlled diabetes	5	21.0 (4.7–37.3)	32.8 (14.5–51.1)	6.3 (2.3–10.2)
Uncontrolled diabetes	51	25.9 (21.6–30.3)	38.0 (31.8–44.3)	5.6 (4.6–6.6)

**Table 2:** Multivariable-adjusted ORs for the association between lead biomarkers and tooth loss.

	OR (95% CI) per tertile of lead biomarker			
	1	2	3	*p* for trend
Tibia				
Lead concentration (μg/g)	≤ 15.0	16.0–23.0	24.0–96.0	
No. in categories of tooth loss^a^	25/61/24	16/52/43	3/51/58	
Models				
Age	1.00	2.00 (1.17–3.42)	3.90 (2.15–7.07)	
Age + smoking variables^b^	1.00	1.91 (1.10–3.32)	3.33 (1.80–6.19)	
Age + smoking variables + other covariates^c^	1.00	1.81 (1.02–3.18)	3.03 (1.60–5.76)	0.001

Patella				
Lead concentration (μg/g)	≤ 22.0	23.0–36.0	37.0–126.0	
No. in categories of tooth loss^a^	25/58/27	14/59/40	5/47/5	
Models				
Age	1.00	1.69 (0.99–2.87)	3.53 (2.00–6.25)	
Age + smoking variables^b^	1.00	1.43 (0.83–2.48)	2.74 (1.51–4.96)	
Age + smoking variables + other covariates^c^	1.00	1.32 (0.75–2.32)	2.41 (1.30–4.49)	0.005

Blood				
Lead concentration (μg/dL)	≤ 4.0	4.2–6.4	7.0–35.0	
No. in categories of tooth loss^a^	20/58/48	11/47/31	13/57/44	
Models				
Age	1.00	0.97 (0.57–1.66)	1.17 (0.70–1.93)	
Age + smoking variables^b^	1.00	0.84 (0.48–1.47)	0.89 (0.53–1.52)	
Age + smoking variables + other covariates^c^	1.00	0.86 (0.49–1.50)	0.88 (0.52–1.50)	0.57

a Categories of tooth loss: 0, 1–8, ≥ 9 missing teeth.

b Smoking status (never, former, and current) and pack-years of smoking.

c Education (< high school, high school, technical training/some college, or college graduate or higher) and diabetes (no, controlled, uncontrolled).
